# Do Muscle Changes Contribute to the Neurological Disorder in Spastic Paresis?

**DOI:** 10.3389/fneur.2022.817229

**Published:** 2022-03-14

**Authors:** Maud Pradines, Mouna Ghédira, Blaise Bignami, Jordan Vielotte, Nicolas Bayle, Christina Marciniak, David Burke, Emilie Hutin, Jean-Michel Gracies

**Affiliations:** ^1^UR 7377 BIOTN, Laboratoire Analyse et Restauration du Mouvement, Université Paris Est Créteil (UPEC), Créteil, France; ^2^AP-HP, Service de Rééducation Neurolocomotrice, Unité de Neurorééducation, Hôpitaux Universitaires Henri Mondor, Créteil, France; ^3^Department of Physical Medicine and Rehabilitation, Northwestern University and the Shirley Ryan AbilityLab, Chicago, IL, United States; ^4^Department of Neurology, Northwestern University and the Shirley Ryan AbilityLab, Chicago, IL, United States; ^5^Department of Neurology, Royal Prince Alfred Hospital and the University of Sydney, Sydney, NSW, Australia

**Keywords:** spastic myopathy, spastic cocontraction, chronic hemiparesis, synaptic sensitization, clinical extensibility, muscle disorder, stretch-sensitive paresis, quantified assessment

## Abstract

**Background:**

At the onset of stroke-induced hemiparesis, muscle tissue is normal and motoneurones are not overactive. Muscle contracture and motoneuronal overactivity then develop. Motor command impairments are classically attributed to the neurological lesion, but the role played by muscle changes has not been investigated.

**Methods:**

Interaction between muscle and command disorders was explored using quantified clinical methodology—the Five Step Assessment. Six key muscles of each of the lower and upper limbs in adults with chronic poststroke hemiparesis were examined by a single investigator, measuring the angle of arrest with slow muscle stretch (X_V1_) and the maximal active range of motion against the resistance of the tested muscle (X_A_). The coefficient of shortening C_SH_ = (X_N_-X_V1_)/X_N_ (X_N_, normally expected amplitude) and of weakness C_W_ = (X_V1_-X_A_)/X_V1_) were calculated to estimate the muscle and command disorders, respectively. Composite C_SH_ (CC_SH_) and C_W_ (CC_W_) were then derived for each limb by averaging the six corresponding coefficients. For the shortened muscles of each limb (mean C_SH_ > 0.10), linear regressions explored the relationships between coefficients of shortening and weakness below and above their median coefficient of shortening.

**Results:**

A total of 80 persons with chronic hemiparesis with complete lower limb assessments [27 women, mean age 47 (SD 17), time since lesion 8.8 (7.2) years], and 32 with upper limb assessments [18 women, age 32 (15), time since lesion 6.4 (9.3) years] were identified. The composite coefficient of shortening was greater in the lower than in the upper limb (0.12 ± 0.04 vs. 0.08 ± 0.04; *p* = 0.0002, while the composite coefficient of weakness was greater in the upper limb (0.28 ± 0.12 vs. 0.15 ± 0.06, lower limb; *p* < 0.0001). In the lower limb shortened muscles, the coefficient of weakness correlated with the composite coefficient of shortening above the 0.15 median C_SH_ (*R* = 0.43, *p* = 0.004) but not below (*R* = 0.14, *p* = 0.40).

**Conclusion:**

In chronic hemiparesis, muscle shortening affects the lower limb particularly, and, beyond a threshold of severity, may alter descending commands. The latter might occur through chronically increased intramuscular tension, and thereby increased muscle afferent firing and activity-dependent synaptic sensitization at the spinal level.

## Introduction

In spastic paresis, muscle changes coexist with neurologic abnormalities (1, 2) and the two have been suggested to potentiate each other (3–5). Disruption of the motor command causes immediate paresis, i.e., reduced voluntary motor unit recruitment, which, in the context of muscle hypo-mobilization, triggers a cascade of pathological changes affecting muscle tissue extensibility and motor neuronal excitability (4, 5).

In the acute stages, the common occurrence of hypo-mobilization of several paretic muscles in a shortened position (6) represents an assault on muscle tissue, causing acute transformation in molecular genetics and gene transcription (4, 5, 7–9). Within days, muscle mass is reduced and muscle extensibility decreases, in parallel with sarcomere loss (10–12); in addition, collagen tissue is modified and deposits around muscle fibers with fascial thickening (13–16). These muscle changes can be demonstrated through biomechanical and clinical measurements; they gradually worsen if hypo-mobilization is not addressed (16–21).

In the subacute stages of spastic paresis, additional plastic neural mechanisms come into play, whereby stretch-sensitive (spastic) muscle overactivity causes a third mechanism to contribute to the motor impairment, along with the agonist paresis and the muscle disorder (4, 5, 21–23). Antagonist muscle overactivity predominates in some muscles, producing agonist-antagonist imbalance around joints (5, 24, 25). Among the various types of muscle overactivity in spastic paresis, spastic co-contraction has been defined as a misdirection of the supraspinal drive that abnormally recruits antagonist motor units during agonist command, independent of any phasic stretch (5, 26, 27). This form of overactivity directly impedes and may sometimes reverse the desired voluntary movement (26). Stretch receptor recruitment in the overactive muscle aggravates this antagonistic co-contraction, hence the term *spastic* co-contraction (26–28). Finally, for the agonist muscles responsible for the movement, the responsiveness of agonist motor neurons to descending command may be negatively impacted by the stretch imposed on the antagonist, producing a *stretch-sensitive paresis* (4, 28, 29).

Decreased extensibility of the antagonist muscle in hemiparesis can be estimated clinically as reduced passive range of motion when attempting maximal slow and strong passive movement against the resistance of the tested muscle group. Here, we question whether decreased muscle extensibility might play a role in the later plastic neural events described above (spastic co-contraction and stretch-sensitive agonist paresis). The second question is whether motor function seems compromised more by the muscle disorder or the abnormal descending command. A better understanding of the respective contributions of muscle and neural disorders to functional impairment in chronic hemiparesis might then indicate where interventions should be mostly directed for individual patients. To answer these questions, we conducted a retrospective investigation of two coefficients of impairment designed as normalized, clinical estimates of the muscle and the neural components of spastic paresis. The overarching hypothesis tested here was that, beyond a threshold of muscle shortening in chronic hemiparesis, the transmission of active command to an agonist may depend, at least in part, on the severity of collagenous modifications of the antagonist, i.e., on the degree of antagonist hypo-extensibility.

## Methods

### Study Design

**Figure 1:**
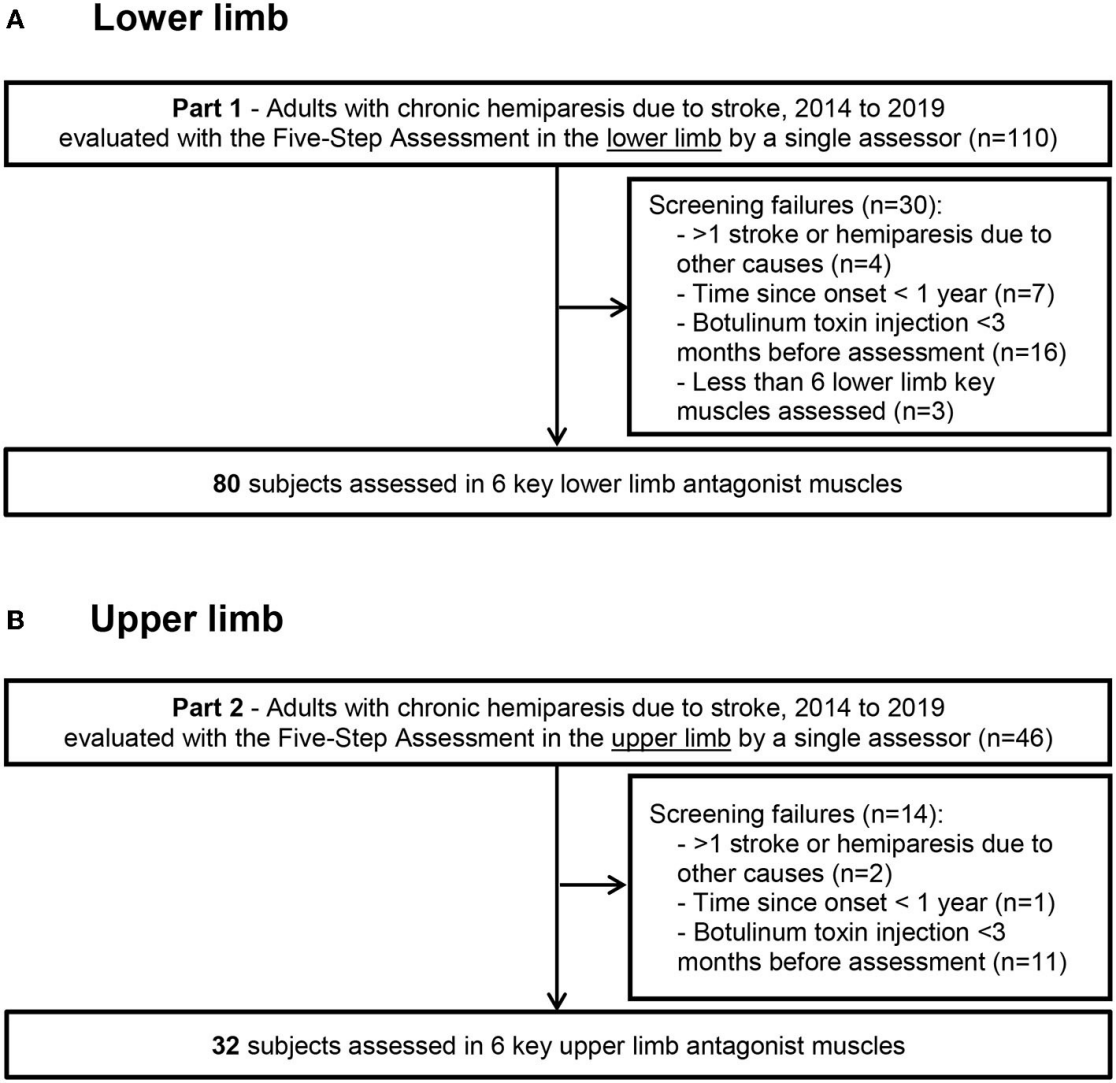
Flow diagram Flow diagram for the lower limb **(A)** and upper limb **(B)** data collection.

This study was conducted in compliance with the Declaration of Helsinki (2008), Good Clinical Practice guidelines, and local regulatory requirements for the Henri Mondor University Hospital, Créteil, France. We retrospectively reviewed the charts of subjects with chronic hemiparesis that had been consecutively evaluated using at least steps 1, 2, and 4 of the Five *Step Assessment* (an expansion of the Tardieu scale, see below) (30) in the lower and/or the upper limb, by a single clinician (JMG) between January 2014 and December 2019 (Figure 1).

### Inclusion and Exclusion Criteria

All the patient charts were included in this study if they fulfilled the following criteria: (i) adults (age ≥ 18 years) with chronic hemiparesis due to a single stroke that occurred > 1 year before the assessment (Figure 1); (ii) assessment using at least Steps 1, 2, and 4 of the Five Step Assessment in soleus (SO), gastrocnemius (GN), gluteus maximus (GM), hamstrings (HS), vastus (VA) and rectus femoris (RF) for the lower limb, or shoulder extensors (SE), subscapularis (SS), pronator quadratus (PQ), elbow flexors (EF), wrist flexors (WF) and finger flexors (FF) for the upper limb, performed between January 2014 and December 2019 (iii) ability for independent ambulation (Functional Ambulation Classification score of 5–6) (31); (iv) absence of botulinum toxin injection in the evaluated muscles in the 3 months before the assessment; and (v) clinically stable condition. Exclusion criteria were: (i) recurrent strokes or other neurological or orthopedic disorders affecting the evaluated muscles; (ii) severe cognitive impairment (Mini-Mental Status test score < 23 or major receptive aphasia) interfering with the ability to assess the patient; (iii) treatment with antispasticity medications that could produce synaptic depression, whether oral or intrathecal (baclofen, benzodiazepines, etc) (32, 33). In each patient chart, the first visit chronologically that met these criteria was selected for analysis.

### Description of the Five Step Assessment

For each subject, the same clinician used the Five Step Assessment for the six muscles defined above in the lower limb and/or the upper limb (30). The patient was always seated for the upper limb assessments and supine for the lower limb assessments (apart from locomotion).

Step 1 of the Five Step Assessment evaluates active function. For the lower limb, this involved measurement of ambulation speed in meters/second (m/s) over 10 m (AT10) performed barefoot at maximal speed with the assessment starting and ending in a seated position (19, 30, 34). For the upper limb, the active function was measured using the Modified Frenchay Scale (MFS), and consisted of videotaping ten activities of daily living (4 uni-manual activities using the paretic hand and 6 bimanual activities, in which the paretic hand assists the other hand) and rating each of them on a ten-point visual analog scale based on video review (19, 35). In that visual analog scale, zero means no movement, 10 is the perfect achievement of the task, and 5 is a task barely accomplished (36). The 10 scores were averaged to derive the MFS score for each patient. Individual task rating on the MFS has excellent intra- and inter-reliability and the MFS has been validated against a subjective scale of perceived function (Disability Assessment Scale, DAS) as well as the Fugl–Meyer score, a classic measurement of motor impairment (19, 36–38).

Step 2 of the Five Step Assessment is the measurement of the *passive range of motion* of the tested muscle, referred to as the X_V1_ angle of the Tardieu scale (19, 30, 39), using zero as the theoretical angle of minimal stretch for each muscle (19, 30, 39). Stretch was applied slowly and strongly, up to the point where further passive stretch was not possible or would cause pain or would jeopardize joint integrity (30, 39). The stretch was performed as slowly as possible to avoid triggering a phasic stretch reflex and as strong as possible to overcome most of the spastic dystonia (30, 40). X_V1_ angle measurements were made visually, as the reliability of these angle measurements has been shown to be similar between visual or goniometric evaluations (41), except at the knee where a goniometer was used. X_V1_ measurements have shown good to excellent intra- and inter-rater reliability in paretic adults (41–44); this measure provides information primarily about the extensibility of *muscle* tissue (45). From the X_V1_ measure, the coefficient of shortening C_SH_ was derived for each muscle (19), based on the formula C_SH_ = (X_N_-X_V1_)/X_N_ where X_N_ is the normally expected passive joint amplitude for each tested muscle (46). The normally expected passive amplitude X_N_ is also defined using zero as the theoretical angle of minimal stretch for each muscle (as in the Tardieu Scale). In this study, the normal reference X_N_ was considered to be 120° for the SO, 115° for GN, 150° for GM, 270° for HS (180° knee extension + 90° hip flexion), 150° for VA muscles, 240° for RF (150° knee flexion + 90° hip extension), 180° for the SE, SS, EF and WF, PQ, and 270° for FF (46, 47).

**Figure 2:**
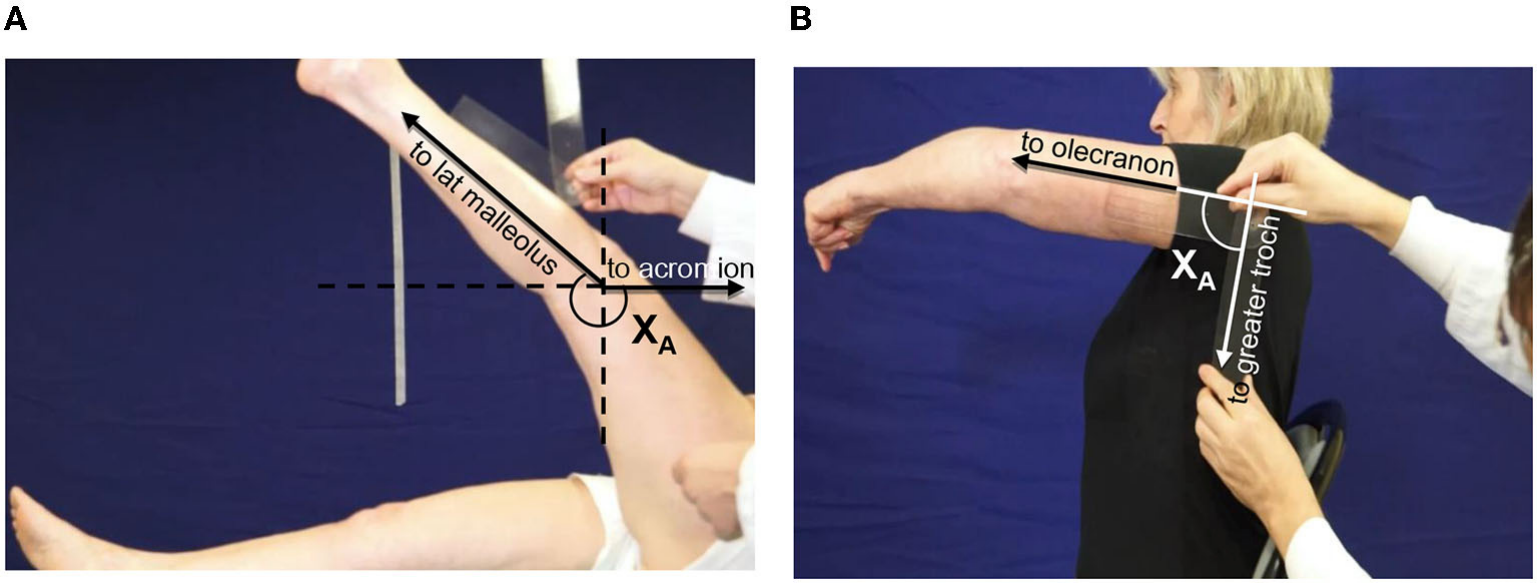
Clinical assessment of active movement X_A_. **(A)** Hamstrings: patient supine, lower limb lying straight on the table. The clinician asks for a hip flexion keeping the knee extended. Axis of rotation: lateral condyle. The angle X_A_ is measured between the two lines lateral condyle-external malleolus and lateral condyle-acromion (through projection, parallel to the line greater trochanter-acromion). **(B)** Shoulder extensors: patient seated, upper limb alongside the body. The clinician asks for a shoulder flexion keeping the elbow extended. Axis of rotation: acromion. The angle X_A_ is measured between the two lines acromion-olecranon and acromion-GT.

Step 4 of the Five Step Assessment is the measurement of the maximal *active range of motion* X_A_, against the resistance of the evaluated antagonist muscle (30). The patient was asked to accomplish one movement of maximal amplitude against the resistance of the tested antagonist; here a goniometer was used to measure the angle (Figure 2). The maximal range of active movement represents the balance between the forces generated by agonist activation and those related to the passive and active resistances generated by the tested antagonist muscle group. From the X_A_ measure, the coefficient of weakness C_W_ was derived for each tested muscle, using the formula C_W_= (X_V1_-X_A_)/X_V1_ (19). The coefficient of weakness estimates the impairment of active command against the resistance of the tested antagonist, its maximal passive extensibility being taken into account (19). The intra- and inter-rater reliabilities of X_A_ and of the coefficient of weakness have been established (44).

### Data Treatment

Over the period from January 2014 to December 2019, data for each (upper/lower) limb of each subject from the first visit that met the inclusion criteria were systematically collected. For each lower and upper limb muscle, we normalized its X_V1_ value to a theoretically expected value of 180°, using the formula X_V1normalized_ = X_V1_ × 180/X_N_. From here on, the symbol X_V1_ represents these normalized X_V1_ values. This normalization allowed a Composite X_V1_ to be derived, by averaging the normalized X_V1_ values for the six key muscle groups of each limb:


$$\begin{array}{l} \text{Composite }X_{\text{V1-LL}}=(X_{\text{V1SO}}+X_{\text{V1GN}}+X_{\text{V1GM}}\\ \;+X_{\text{V1HS}}+X_{\text{V1VA}}\;+X_{\text{V1RF}})/6;\\ \text{Composite }X_{\text{V1-UL}}=(X_{\text{V1SE}}+X_{\text{V1SS}}+X_{\text{V1EF}}\\ \;+X_{\text{V1PQ}}+X_{\text{V1WF}}+X_{\text{V1FF}})/6. \end{array}$$

Composite X_A_ for each limb was calculated using the same process (48). We then calculated a *composite coefficient of shortening* as (180-Composite X_V1_)/180 and a *composite coefficient of weakness* as (Composite X_V1_-Composite X_A_)/Composite X_V1_.

Finally, for the shortened muscle groups defined as those with a mean C_SH_ > 0.10, two subgroups were considered: those below and those above their median coefficient of shortening.

### Statistical Analysis

Descriptive statistics were used for the parameters X_V1_ and X_A_ for each muscle, Composite X_V1_, Composite X_A_, coefficient of shortening and coefficient of weakness per muscle, composite, ambulation speed, and Modified Frenchay Score. *t*-tests for paired data were used to compare the coefficients of shortening between individual muscles. Univariate logistic regression analyses then explored correlations between the Composite X_V1_ and the Composite X_A_ (raw data) and between the coefficient of shortening and the coefficient of weakness (normalized data) for each individual muscle group, for the mean of the six muscles of each limb (composite scores) and for the most shortened muscle groups only (C_SH_ > 0.10). In those muscles, correlations between the coefficient of shortening and the coefficient of weakness were explored in the total sample and in the two subgroups below or above the median coefficient of shortening.

Secondarily, univariable regression analyses explored, for each muscle group of the upper and lower limb and for the composite scores, the respective impacts of the coefficient of shortening and of the coefficient of weakness on motor function (Modified Frenchay score or maximal ambulation speed). If both the coefficient of shortening and the coefficient of weakness were predictive of motor function on univariable analyses, bivariable regression analysis was used, for each individual muscle and for the composite score. Lastly, for each limb, if the coefficients of shortening of several muscle groups were predictive of motor function in univariable analyses, multivariable regression explored which of these muscles remained important determinants of motor function once the regression coefficients were adjusted for the effects of the other predictors. Statistical significance was set at 0.01. All analyses were conducted with SPSS (18.0) software.

## Results

### Subject Characteristics

Of 110 consecutive patients with adult-onset chronic hemiparesis in whom the lower limb was evaluated during the study period, 80 patients met the criteria for inclusion (aged 51 ± 16 years; 26 women, 54 men; 42 injured on the left hemisphere, 58 right hemisphere; 66 with ischemic stroke, time since lesion, 9 ± 8 years; Figure 1). For the upper limb group, 46 consecutive patients with adult-onset chronic spastic paresis were evaluated during the study period and 32 patients met the inclusion criteria (aged 39 ± 15 years; 18 women, 14 men; 19 injured on the left hemisphere, 13 on the right; 22 with ischemic stroke, time since lesion, 6 ± 9 years). In 15 of these 32 patients, investigations were performed on both the upper limb and the lower limb. At the selected visit, the Modified Frenchay score was 5.47 ± 1.09 for the upper limb cohort (normal function = 10), and the maximal ambulation speed barefoot was 0.88 ± 0.39 m/s for the lower limb cohort (normal around 1.7 m/s) (34).

### Muscle Shortening and Motor Command Disorder

**Figure 3:**
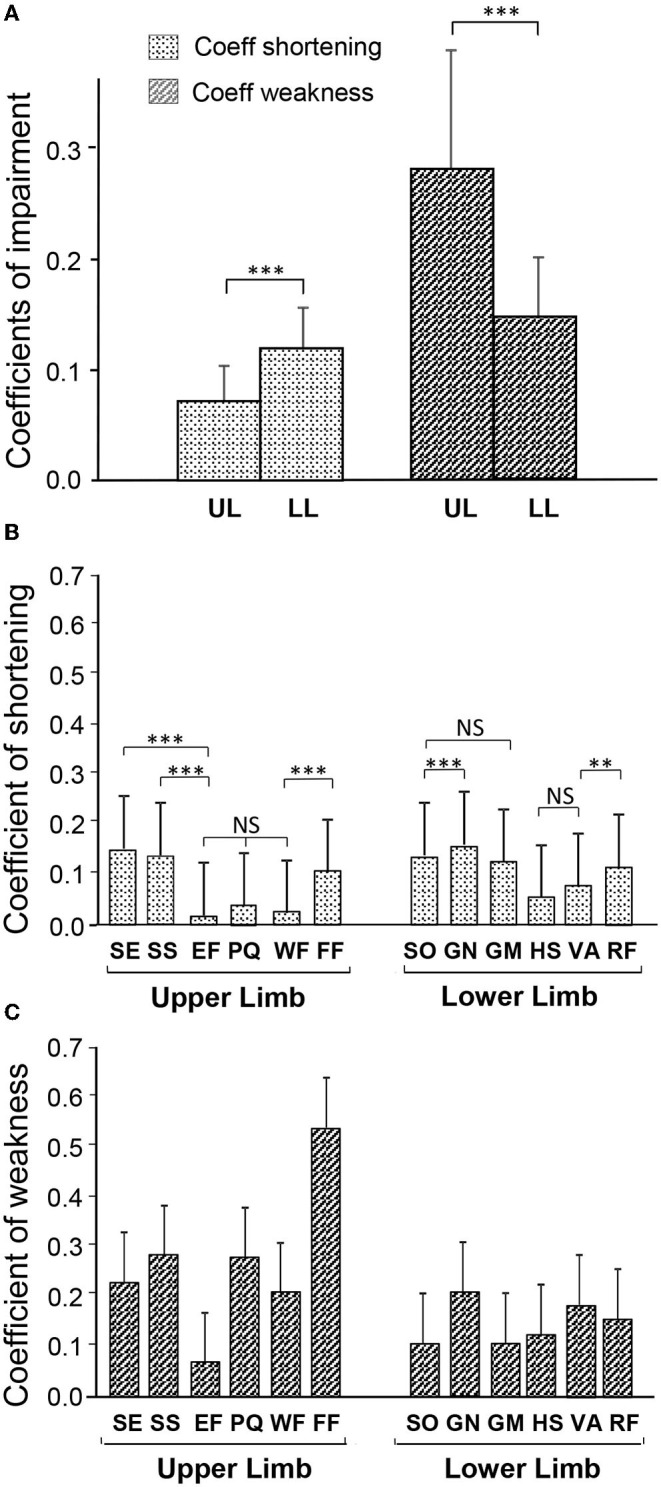
Degree of muscle shortening and of motor command impairment in chronic hemiparesis. **(A)** Coefficients of impairment of the Composite score of the upper and lower limbs. **(B)** Coefficient of Shortening of each of the investigated muscles. **(C)** Coefficient of Weakness of each of the investigated muscles. SE, shoulder extensor; SS, subscapularis; EF, elbow flexors; PQ, pronatus quadratus; WF, wrist flexors; FF, finger flexors; SO, soleus; GN, gastrocnemius; GM, gluteus maximus; HS, hamstrings; VA, vastus; RF, rectus femoris. **p < 0.01; ***p < 0.001.

Overall, the coefficient of shortening (C_SH_) was greater in the lower limb than in the upper limb [0.12, CI 95 (0.11–0.13) vs. 0.08 (0.07–0.09), upper limb; *p* = 0.0002, *t*-test; see Figure 3A, Table 1]. Conversely, the coefficient of weakness (C_W_) was greater in the upper than in the lower limb [0.28 (0.24–0.32) vs. 0.15 (0.14–0.16) lower limb; *p* < 0.0001, *t*-test]. These relationships were similar in the 15 patients in whom there was documentation for both the upper and lower limbs [C_SH_ lower limb 0.11 (0.085; 0.133) vs. upper limb 0.08 (0.055; 0.097); *p* = 0.03 *t*-test; C_W_ upper limb 0.35 (0.277; 0.423) vs. lower limb 0.16 (0.125; 0.195); *p* = 0.0015; not illustrated].

**Table 1:** Clinical parameters and comparison of muscle shortening.

**A. Clinical parameters**													
**Lower limb**	**Soleus**	**Gastroc**	**Glut Max**	**Hamst**	**Vastus**	**Rect Fem**	**Comp score**						
X_V1_	102 ± 6	95 ± 4	129 ± 12	253 ± 13	137 ± 7	208 ± 16	154 ± 7						
X_A_	91 ± 9	76 ± 13	116 ± 16	222 ± 23	112 ± 16	176 ± 25	132 ± 13						
C_SH_	0.15 ± 0.05	0.17 ± 0.04	0.14 ± 0.08	0.06 ± 0.05	0.09 ± 0.05	0.13 ± 0.07	0.12 ± 0.04						
C_W_	0.10 ± 0.07	0.21 ± 0.12	0.10 ± 0.07	0.12 ± 0.08	0.18 ± 0.11	0.15 ± 0.09	0.15 ± 0.06						
Ambul speed	0.88 ± 0.39												
**Upper limb**	**Sh Ext**	**Subscap**	**Elbow flex**	**Pron quad**	**Wrist flex**	**Finger flex**	**Comp score**						
X_V1_	150 ± 22	152 ± 23	177 ± 4	177 ± 16	175 ± 6	264 ± 13	183 ± 15						
X_A_	116 ± 33	111 ± 37	165 ± 13	128 ± 44	138 ± 21	121 ± 63	130 ± 11						
C_SH_	0.16 ± 0.12	0.15 ± 0.13	0.01 ± 0.02	0.04 ± 0.08	0.03 ± 0.03	0.12 ± 0.04	0.08 ± 0.04						
C_W_	0.23 ± 0.18	0.29 ± 0.17	0.07 ± 0.06	0.28 ± 0.23	0.21 ± 0.11	0.54 ± 0.25	0.28 ± 0.12						
MFS	5.5 ± 1.1												
**B. Differences between coefficients of shortening of individual muscles**													
**Upper Limb**							**Lower Limb**						
	C_SH-SE_	C_SH-SS_	C_SH-EF_	C_SH-QP_	C_SH-WF_	C_SH-FF_		C_SH-SOL_	C_SH-GAS_	C_SH-GM_	C_SH-HS_	C_SH-VA_	C_SH-RF_
C_SH−SE_							C_SH−SOL_						
C_SH−SS_	0.55						C_SH−GAS_	3E-06					
C_SH−EF_	8E-08	2E-06					C_SH−GM_	0.011	3E-04				
C_SH−QP_	2E-06	4E-06	0.053				C_SH−HS_	3E-15	6E-26	1E-10			
C_SH−WF_	9E-08	2E-06	0.03	0.31			C_SH−VA_	2E-11	2E-19	2E-06	0.013		
C_SH−FF_	0.11	0.17	2E-15	3E-06	4E-11		C_SH−RF_	0.22	2E-03	6E-01	9E-07	2E-04	

*X_V1_, Maximal passive range of motion against the resistance of the investigated muscle; X_A_, active range of motion against the resistance of the investigated muscle; C_SH_, coefficient of shortening; C_W_, coefficient of weakness; MFS, Modified Frenchay Scale; Ambul Speed, ambulation speed over 10 meters (AT10) at maximal speed, barefoot*.

*B. Comparison of muscle shortening (C_SH_) between individual muscles within each limb*.

*SE, shoulder extensor; SS, subscapularis; EF, elbow flexors; PQ, pronatus quadratus; WF, wrist flexors; FF, finger flexors; SO, soleus; GN, gastrocnemius; GM, gluteus maximus; HS, hamstrings; VA, vastus muscles; RF, rectus femoris*.

The individual coefficients of shortening, and of weakness, for the six muscles in each limb are displayed in Figures 3B,C, respectively. In the lower limb, over the six evaluated muscles, GN exhibited the greatest shortening: C_SH−GN_ = 0.17 (0.16; 0.18). Then SO: C_SH−SO_ = 0.15 (0.14; 0.16), GM C_SH−GM_ = 0.14 (0.06; 0.22) and RF C_SH−RF_ = 0.13 (0.06; 0.20) were shortened to about the same extent (Table 1B; Figure 3B). In the upper limb, the three most shortened muscles were SE: C_SH−SE_ = 0.16 (0.12; 0.20), SS: C_SH−SS_ = 0.15 (0.10; 0.18), and FF: C_SH−FF_ = 0.10 (0.06; 0.14) (Table 1B; Figure 3B). GN: C_W−GN_ = 0.21 (0.18; 0.24) and FF: C_W−FF_ = 0.54 (0.45; 0.63), were exhibiting the highest coefficient of weakness at the lower and upper limb, respectively (Figure 3C).

### Relation Between Coefficients of Shortening and Coefficients of Weakness

**Figure 4:**
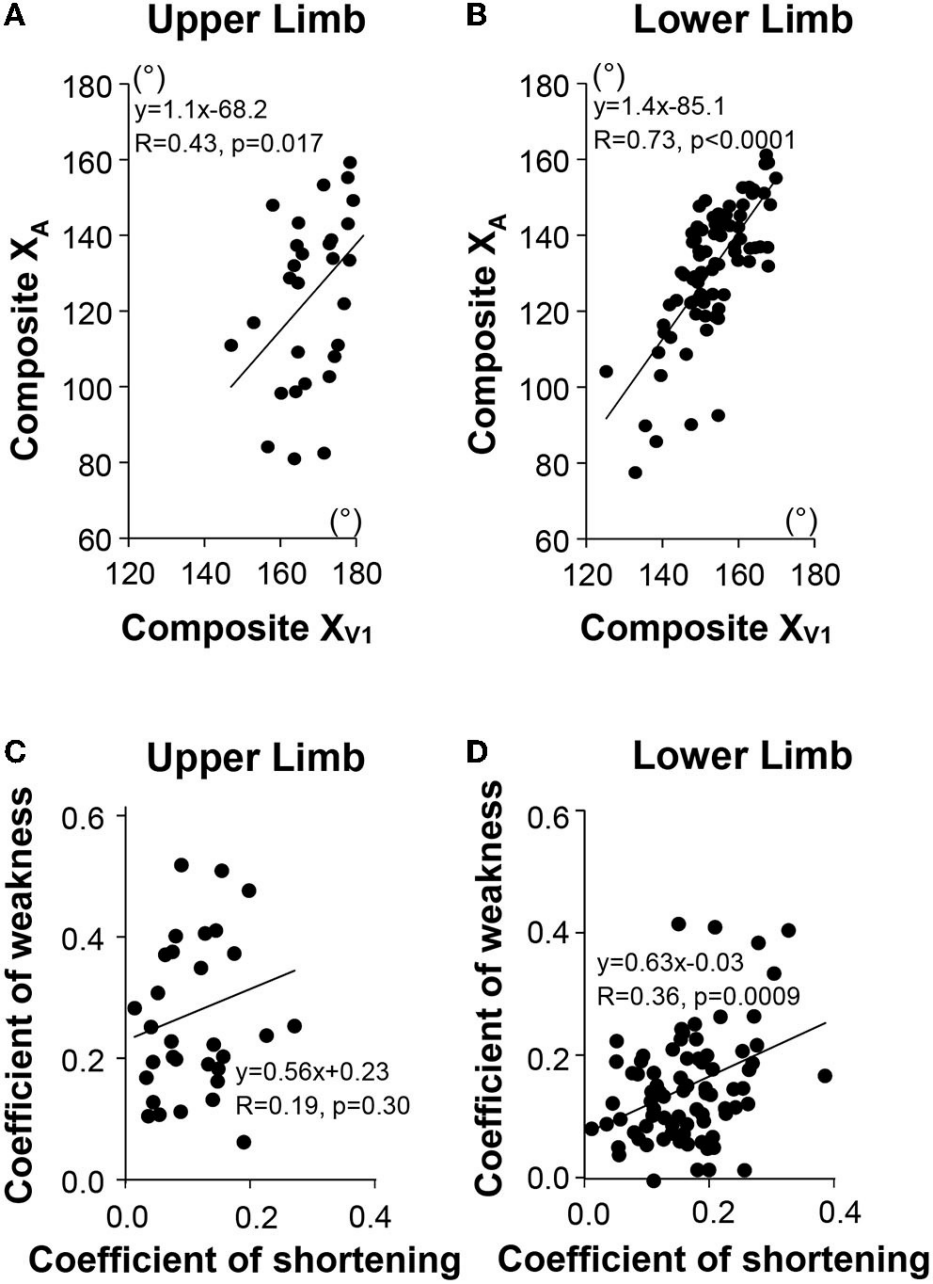
Relationship between passive (X_V1_) and active movements (X_A_) for the upper limb **(A)**, for the lower limb **(B)**. Composite X_V1_, mean X_V1_ of the six investigated muscles; Composite X_A_, mean X_A_ of the six investigated muscles. Relationship between the mean Coefficient of Shortening and the mean Coefficient of Weakness (Composite Scores) in the upper limb **(C)**, in the lower limb **(D)**, respectively.

In terms of raw values in the upper and lower limbs, the maximal active range of motion against the resistance of the examined muscle (Composite X_A_) correlated with the angle of arrest with slow muscle stretch (Composite X_V1_), the correlation being strong in the lower limb (upper limb, *R* = 0.43, *p* = 0.017; lower limb *R* = 0.73, *p* < 0.0001; univariable analysis; see Figure 4). After normalizing and taking the mean coefficients across the six muscles, the composite coefficient of weakness still correlated with the composite coefficient of shortening for the lower limb (*R* = 0.36, *p* = 0.0009). Evaluation of each muscle group individually found this dependency in the GN only (*R* = 0.36, *p* < 0.0001), with trends in the GM (*R* = 0.22, *p* = 0.045) and HS (*R* = 0.21, *p* = 0.052). In the upper limb, however, the composite coefficient of weakness did not correlate with the composite coefficient of shortening (*R* = 0.19, *p* = 0.30). A trend for this correlation was found only in SS, when examined by individual muscles (*R* = 0.48, *p* = 0.016).

**Figure 5:**
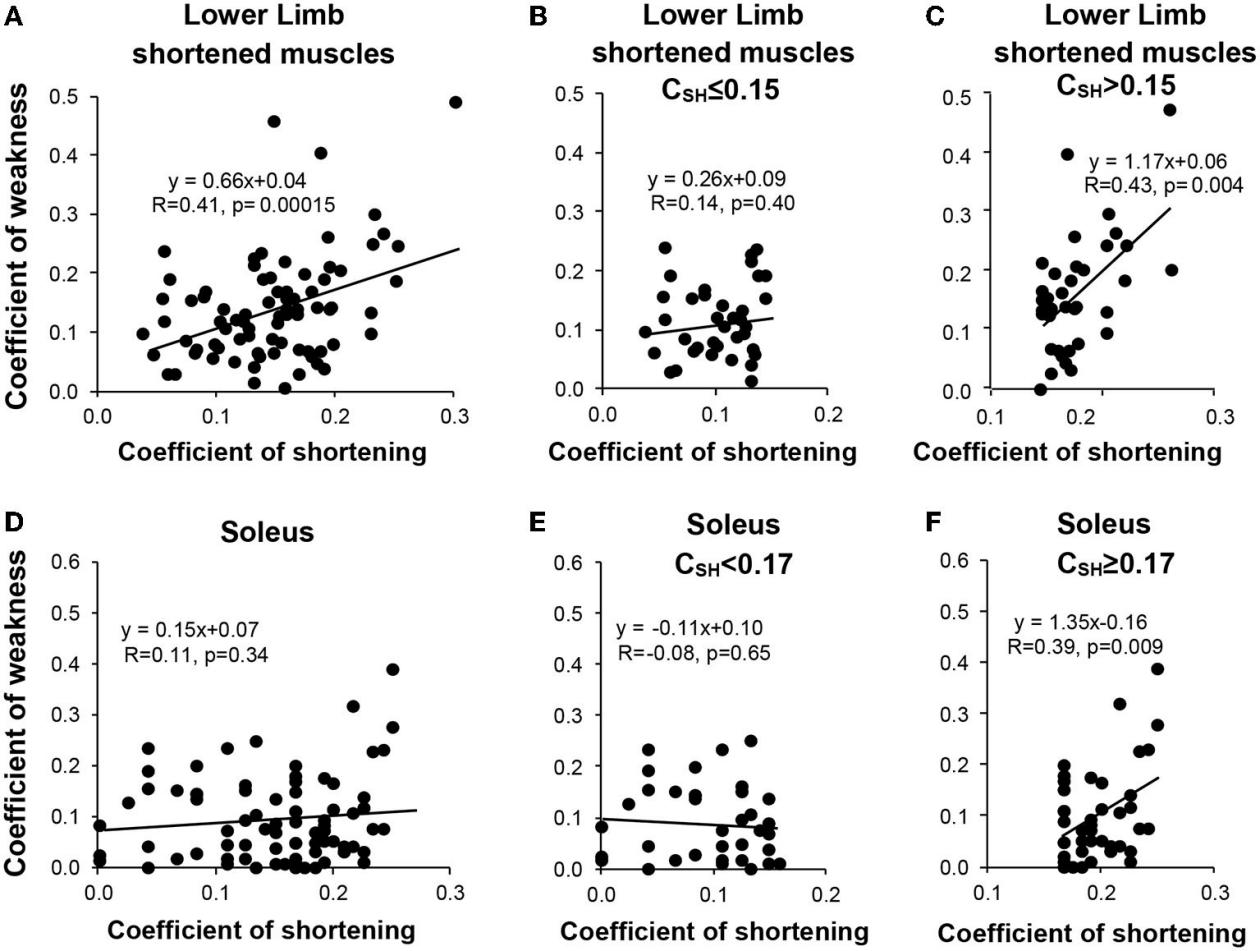
Relationship between the muscular (shortening) and the neurological (weakness) disorders for the shortened muscle groups of the lower limb (sample of the muscle groups with mean CSH > 0.10, i.e., soleus, gastrocnemius, gluteus maximus, and rectus femoris) **(A–C)** and for the soleus muscle **(D–F)**. Left, the entire sample **(A,D)**; middle, coefficients of shortening below the median **(B,E)**; right, coefficients of shortening beyond the median **(C,F)**.

Univariable analysis for the most shortened muscles of the lower limb taken together (Figures 5A–C) (i.e., those muscles with C_SH_ > 0.10, which was the case for SO (Figures 5D–F) GN, GM, and RF), showed that C_SH_ correlated with the coefficient of weakness for patients with values above the median (Figure 5B), but not for patients with coefficients of shortening below the median (Figure 5C). This was also true for the SO (Figures 5E,F) and GN (not shown), taken individually.

### Relationships of the Coefficients of Shortening and Weakness With Motor Function

**Figure 6:**
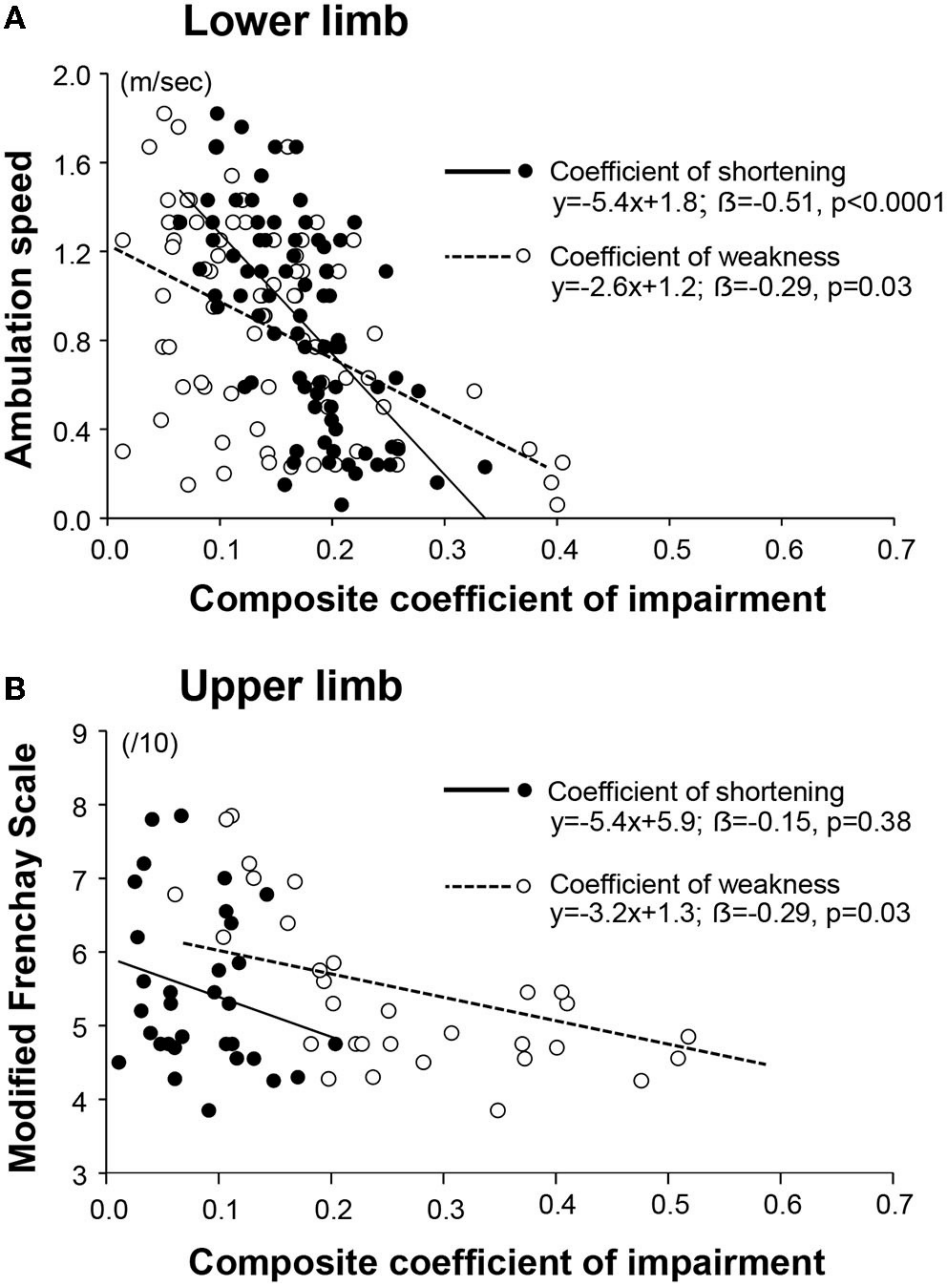
Respective contribution of the muscular and the neurological disorders to functional impairments. The coefficient of impairment refers to the coefficient of shortening or the coefficient of weakness, as indicated in the legend. The composite coefficient is the mean of the individual coefficients for each of the six muscles of the lower limb **(A)**; and of the upper limb **(B)**.

In univariable analyses for the lower limb, each of the composite coefficients of shortening (strongly) and of weakness predicted motor function (*R* = −0.62, *p* < 0.0001; *R* = −0.48, *p* < 0.0001, respectively, Table 2A). When exploring the impact of the extensibility loss in each of the six lower limb muscles taken individually, ambulation speed depended on the coefficient of shortening primarily (Table 2A). In the upper limb, the Modified Frenchay score depended on coefficients of weakness only (Figure 6B, Table 2A).

**Table 2:** Respective contribution of the muscular and the neurological disorders to functional impairments.

**A. Univariable analyses: for C**_**SH**_ **and C**_**W**_**, respectively**															
**Lower limb**	**Pred Fact**	**Soleus**		**Gastroc**		**Glut Max**		**Hamst**		**Vastus**		**Rect Fem**		**Comp score**	
		***r***	***p***	***r***	***p***	***r***	***p***	***r***	***p***	***r***	***p***	***r***	***p***	***r***	***p***
Max ambul Speed	C_SH_	−0.33	0.004	−0.39	<0.001	−0.50	<0.001	−0.58	<0.001	−0.51	<0.001	−0.45	<0.001	−0.62	<0.001
	C_W_	−0.37	<0.001	−0.48	<0.001	−0.31	0.006	−0.30	0.008	−0.41	<0.001	−0.16	0.17	−0.48	<0.001
**Upper Limb**	**Pred Fact**	**Sh Ext**		**Subscap**		**Elbow Flex**		**Pron Quad**		**I: Wrist Fex**		**I: Finger Fex**		**Comp score**	
		***r***	***p***	***r***	***p***	***r***	***p***	***r***	***p***	***r***	***p***	***r***	***p***	***r***	***p***
MFS (Frenchay)	C_SH_	−0.32	0.08	−0.31	0.12	0.30	0.10	−0.11	0.54	0.01	0.95	0.18	0.26	−0.22	0.21
	C_W_	−0.48	0.008	−0.27	0.20	−0.37	0.04	−0.48	0.006	−0.53	0.002	−0.69	<0.001	−0.43	0.013
**B. Bivariable analyses: impact of C**_**SH**_ **and C**_**W**_ **(taken together) on motor function when for a given muscle, each of the two coefficients (C**_**SH**_ **and C**_**W**_**)**																
**was individually correlated to motor function**																
**Lower limb**	**Pred Fact**	**Soleus**		**Gastroc**		**Glut Max**		**Hamst**		**Vastus**		**Rect Fem**		**Comp score**	
		**β**	***p***	**β**	***p***	**β**	***p***	**β**	***p***	**β**	***p***	**β**	***p***	**β**	***p***
Max ambul Speed	C_SH_	−0.33	0.002	−0.22	0.045	−0.45	<0.001	−0.54	<0.001	−0.49	<0.001	–	–	−0.51	<0.001
	C_W_	−0.37	<0.001	−0.39	<0.00	−0.20	0.047	−0.19	0.049	−0.38	<0.001	-	–	−0.29	0.003
**Upper Limb**	**Pred Fact**	**Sh Ext**		**Subscap**		**Elbow Flex**		**Pron Quad**		**I: Wrist Fex**		**I: Finger Fex**		**Comp score**	
		**β**	***p***	**β**	***p***	**β**	***p***	**β**	***p***	**β**	***p***	**β**	***p***	**β**	***p***
	C_SH_	−0.34	0.03	–	–	–	–	–	–	–	–	–	–	–	–
	C_W_	−0.46	0.008	–	–	–	–	–	–	–	–	–	–	–	–

*C_SH_, coefficient of shortening; C_W_, coefficient of weakness; MFS, Modified Frenchay Scale; Max Ambul Speed, ambulation speed over 10 meters (AT10) at maximal speed, barefoot*.

In the bivariable analysis for the lower limb, the composite coefficient of shortening and the composite coefficient of weakness remained both predictors of maximal ambulation speed once the regression coefficients were adjusted for the effect of the other predictor, although the coefficient of shortening remained a stronger predictor (composite coefficient of shortening β= −0.51, *p* < 0.0001; coefficient of weakness β = −0.29; *p* = 0.003; Figure 6A, Table 2B). In multivariable analysis, none of the six evaluated lower limb muscles remained a sole predictor of maximal ambulation speed once the regression coefficients were adjusted for the effects of the other muscles.

## Discussion

The present findings suggest that, in chronic hemiparesis, abnormal muscle properties reach greater severity and contribute more to disability in the lower limb than in the upper limb, while in contrast, the upper limb is affected more by the severity of the impairment of the descending command. In the more shortened lower limb muscles (SO, GN, RF, and GM), the coefficient of weakness correlated with the coefficient of muscle shortening above the median coefficient of shortening only. In the upper limb, a trend for such correlation was present for SS only, one of the most shortened upper limb muscles. In chronic spastic paresis, a tipping point may exist where, past a certain threshold of severity, muscle shortening starts worsening the disordered descending command.

### Value of a Clinical Methodology Such as the Five Step Assessment to Draw Inferences Into Actual Muscle Shortening and Actual Impairment of the Descending Command

The Five Step Assessment represents a clinical attempt to distinguish between the muscle disorder and the neurological disorder of spastic paresis, using specific coefficients of impairment (19, 30). However, there is a “double nature” in coefficients of shortening and of weakness, both combining muscular and neural components, that should be discussed.

As for passive X_V1_ measurements (passive range of motion against the tested muscle), when stretching a muscle slowly in a patient at rest, one faces the classic impossibility to safely distinguish between residual spastic dystonia (a neural component of stiffness) and true, passive muscle hypo-extensibility (muscular component) at the end of the passive range (40). Yet, it is commonly accepted by the clinical community that the limitations encountered during maximal passive movements are, for the most part, linked to muscle shortening rather than to residual dystonia. Indeed, except possibly for very large muscles (GM), it is likely that the hand of the examiner typically manages to overcome most of the muscle activation due to residual dystonia. On the other hand, the examiner's hand is bound to fail to overcome all of the passive resistance, as that resistance to passive movement is known to increase exponentially with the amount of stretch in a resting muscle; therefore, passive resisting force is bound to become greater than the maximal force developed by the examiner (49, 50). Thus, while the respective contributions of passive and active resistance at the end of the available range are difficult to clinically quantify, passive phenomena constitute the primary source of range limitation, as demonstrated through X_V1_ measurements after lidocaine blocks that still remain far from the expected physiological values (X_N_) (51).

Reciprocally, active X_A_ measurements also partially depend on the passive resistance that the agonist has to overcome along the way. Yet, the agonist muscle is rarely able to achieve the amplitude reached by the hand of the examiner, therefore X_A_ is almost invariably lower than X_V1_, and frequently far lower (52). This means that the agonist recruitment-induced torque cannot make the limb segment reach the maximal passive range that would be allowed by the antagonist, so that X_A_ often finds itself far below “exponential” levels of passive antagonist resistance. Therefore, it is likely—even though difficult to clinically demonstrate, except by the marked increase in active range motion observed after lidocaine blocks (51)—that once arrived at that “submaximal” X_A_ level, the agonist recruitment is mostly limited by active antagonist co-contractions more than by passive antagonist hypo-extensibility.

Overall, once it is accepted that C_SH_ and C_W_ represent substantially passive vs. active phenomena through these X_V1_ and X_A_ clinical estimates, why would the relationship between them vary depending on X_V1_ or on the limb (upper vs. lower) from which one selects muscles? In other words, if both X_V1_ (maximal passive movements) and X_A_ (maximal active movements) manoeuvers were to be hindered in constant proportions by passive and active components, the correlation between the coefficients of shortening and the coefficients of weakness would be constant, whichever the values of X_V1_. The fact that the correlation emerges only beyond a certain threshold value of X_V1_ is a strong suggestion—if not a demonstration—that muscle shortening itself is associated with further deterioration of the quality of the descending command (e.g., by causing increased co-contraction).

### Causal Relationship or “Casual” Association Between Muscle Shortening and Weakness of Motor Command

Although the correlations do not prove causality by themselves, the respective time courses between the muscle disorder and motoneuronal overactivity, the strength of the correlations above a certain threshold and physiological plausibility suggest a causal relationship, as developed below. Interestingly, it has already been demonstrated that ameliorating the changes in the properties of muscle and soft tissue that constitute the “spastic myopathy” (19) is accompanied by gains in function in the lower limb (53).

### Chronology: The Muscle Disorder Precedes Motoneuronal Overactivity

A substantial body of biological and histological evidence from animal models with limb immobilization demonstrates very early qualitative and quantitative changes in muscle protein synthesis, measured within hours of immobilization, long before any detectable muscle overactivity (7, 8, 11). This chronology has been confirmed in patients with severe hemiparesis from biomechanical measurements, where the onset of passive tissue stiffness of WF was detected long before true neural reflexive stiffness (21).

### Physiological Plausibility: Can Muscle Shortening Aggravate the Abnormal Motor Command?

In models of muscle immobilization in a shortened position in *healthy* animals, muscle spindle firing pathologically increases as shortening of the muscle develops (54–56). These findings were not evident from human microneurography data in spastic paresis, but those studies had insufficient numbers to reach such conclusions (57) or recorded from muscles that were not immobilized in a shortened position [elbow and wrist extensors in Wilson et al. (58) and ankle dorsiflexors in Macefield et al. (59)]. More recently, neuromusculoskeletal models in individuals with chronic stroke-induced hemiparesis suggest that absolute muscle fiber length plays a significant role in the spastic reflex response to imposed movements (60–63). Accordingly, stretch reflex hypersensitivity may be occurring partly through muscle length and extensibility changes, as the pulling force is transmitted more readily to spindles through stiffer hyperelastic structures (4, 5, 54, 56, 64).

A greater muscle afferent input could lead to activity-dependent synaptic plasticity at the spinal level. In chronic hemiparesis, a permanent increase in muscle afferent feedback from the shortened muscle (54, 56) could lead to chronic synaptic sensitization at homonymous α-motoneurons (64–66), decreasing the firing thresholds of these target α-motoneurons. For the antagonist motoneuron pool, a reciprocal situation may occur where activity-dependent synaptic plasticity (66) could sensitize inhibitory synapses in the reciprocal inhibitory pathway, and this would in turn result in increased inhibition of that antagonist motor neuron (and thereby stretch-sensitive paresis). For both the agonist and antagonist motoneuron pools, such facilitatory, respiratory inhibitory, influence from peripheral afferents, might be unleashed by the potentially decreased presynaptic inhibition linked to disengaging supraspinal control after central lesions (67–70). Interestingly, it has been found in animals that remobilization through step-training may restore reciprocal presynaptic inhibition (71).

### Threshold of Effect: Level of Severity Beyond Which Muscle Shortening Might Worsen the Abnormal Motor Command

A relationship between the clinical estimates of shortening and of weakness was observed in the more shortened lower limb muscles, beyond the median value of the composite coefficients of shortening only. The same relationship was not found below the median value of the composite coefficients of shortening in the lower limb or in the upper limb, except for a trend noted for the SS muscle. The low level of muscle shortening (8%) in the upper limb may have been insufficient to create significant muscle afferent firing and synaptic sensitization at the spinal level. On the other hand, the mean shortening found in the lower limb (12%) may be sufficient to reveal this relationship with the clinical tools used here. When looking at individual examples, several muscles fit with this hypothesis: the EF and the WF were characterized by only 2 and 4% of extensibility loss, respectively, and we did not observe any dependence of the neural command on their shortening. In contrast, the extensibility loss was 15% in SS and data did suggest this relationship for this muscle.

### Impact of Passive Mechanical Properties on Active Function

This study confirmed that, in each of the six investigated muscles of the lower limb, the loss of muscle extensibility and impairment of the motor command both affect motor function in spastic paresis. Considering the composite scores of the lower limb, muscle shortening correlated with ambulation speed more strongly than did the estimated neural command component, with a steeper slope, explaining 26% of the variance of walking speed. Furthermore, individual correlations between each of the six coefficients of muscle shortening in the lower limb and motor function in multivariable analysis indicate the need to consider *all* of these six antagonist muscles to swing phase for assessment, and potentially for treatment. None of the lower limb antagonists alone predicted lower limb function once the regression coefficients were adjusted for the effects of the other predictors.

This strong dependence of motor function on mechanical impairment in the lower limb corroborates previous results. For example, passive resistance of the plantar flexors significantly affects active dorsiflexion amplitude during the swing phase in subjects with hemiparesis (1, 72). Similarly, in adults with cerebral palsy muscle shortening starts in early life, is more severe than in acquired paresis and correlates with walking speed, stair ascent, and descent speeds (73–76). In the upper limb, even though the composite coefficient of shortening did not correlate with motor function in this study, ultrasound measured average fascicle length in EF correlated with impairment level in the upper limb of stroke subjects in recent reports (77, 78).

### The Predominance of Muscle Shortening in the Lower Limb and of Primary Motor Command Impairment in the Upper Limb

Muscle shortening was 50% more severe in the lower than in the upper limb. In contrast, the composite coefficient of weakness in the upper limb was almost double that in the lower limb.

The overall mild coefficients of shortening found in the upper limb muscle groups confirm previous results obtained in large international studies also using the Five Step Assessment (46, 52, 79). In the lower limb, GN had the highest coefficient of shortening in this study, similar to values found in previous large studies (80, 81). Such findings reflect the dramatic changes in muscle structure known from previous biomechanical investigations in the hemiparetic lower limb, including reduced pennation angle, shorter fascicle length, decreased passive dorsiflexion, and increased ankle stiffness (82–87).

Greater impairment of the motor command occurred in the upper limb even though there was less muscle shortening. This may indicate that muscle shortening may be only one determinant of the disorder of descending command, obvious other factors being the location and size of the lesion, and the amount of limb disuse since the lesion. Specifically, there is likely to be more severe disuse of the paretic upper limb (also called “functional motor amnesia” or “learned non-use”) (88–90), than of the lower limb. Walking represents a critical daily activity necessarily involving both legs while the use of one upper limb might be partly offset when the other functions normally.

### Clinical Implications

The understanding of the role of the muscle disorder on the neural command and on motor function should encourage therapists to consider the muscle disorder as a nosologic entity in hemiparesis and to implement meaningful therapeutic interventions specifically on this target (21, 91). Descriptive results found in this study will help to direct these interventions, addressing particularly the plantar flexors, GM and RF in the lower limb, and the shoulder muscles and FF in the upper limb. Besides the central nervous system, skeletal muscle is another plastic tissue with response to changes in stimulation and in the environment (18, 91–93). Spastic myopathy should be treated with an appropriate physical treatment, using techniques such as prolonged daily self-stretch postures at high load (53), active stretching (94), short wave and ultrasound therapies (95), as it is known that botulinum toxin injections alone will not allow any long-term meaningful muscle lengthening (52, 96, 97). To minimize muscle damage from the acute stages, vibrations (98), stretching postures through positioning (99, 100) and specific splinting (101), or Leucine and vitamin D (102, 103) might also be helpful.

### Study Limitations

As considered above in the first section of this discussion, the main limitation of this study lies in the clinical nature of its methodology, which comes short of physiologically assessing “true” descending command and “true” passive muscle extensibility. In addition, the weight of the limb segment to lift up against gravity could have impacted the measure of X_A_ particularly against the resistance of three of the investigated muscles (SE, GM, and HS). In addition, brain imaging and the role of lesion locations were not analyzed in this study. Lesion locations may have factored into the observed discrepancy between upper and lower limb features. Also, patients were supposed to be affected by purely singular strokes, but some of them, through a long post-stroke state, might have acquired additional subclinical strokes.

*In conclusion*, increased attention should be directed toward abnormal muscle properties in chronic hemiparesis, particularly in the lower limb in which muscle shortening is severe and harmful to ambulation. Beyond a threshold of severity, the passive biomechanical and structural abnormalities of the most affected muscles may make a significant contribution to the neural command disorder and to functional disability.

## Data Availability Statement

The original contributions presented in the study are included in the article/supplementary material, further inquiries can be directed to the corresponding author/s.

## Ethics Statement

Ethical review and approval was not required for the study on human participants in accordance with the local legislation and institutional requirements. Written informed consent for participation was not required for this study in accordance with the national legislation and the institutional requirements. Written informed consent was obtained from the individual(s) for the publication of any potentially identifiable images or data included in this article.

## Author Contributions

J-MG and MP were involved in the conception and design of the study, in the acquisition and analysis of data, and the draft of the manuscript and figures. CM, DB, JV, BB, NB, EH, and MG participated in the analysis of data, the draft of the manuscript, and checked the final draft of the manuscript. All authors read and approved the final manuscript.

## Conflict of Interest

The authors declare that the research was conducted in the absence of any commercial or financial relationships that could be construed as a potential conflict of interest.

## Publisher's Note

All claims expressed in this article are solely those of the authors and do not necessarily represent those of their affiliated organizations, or those of the publisher, the editors and the reviewers. Any product that may be evaluated in this article, or claim that may be made by its manufacturer, is not guaranteed or endorsed by the publisher.
